# Antagonistic Roles of *SEPALLATA3*, *FT* and *FLC* Genes as Targets of the Polycomb Group Gene *CURLY LEAF*

**DOI:** 10.1371/journal.pone.0030715

**Published:** 2012-02-17

**Authors:** Manuel Lopez-Vernaza, Suxin Yang, Ralf Müller, Frazer Thorpe, Erica de Leau, Justin Goodrich

**Affiliations:** Institute for Molecular Plant Sciences, School of Biology, University of Edinburgh, Edinburgh, United Kingdom; Instituto de Biología Molecular y Celular de Plantas, Spain

## Abstract

In Arabidopsis, mutations in the Pc-G gene *CURLY LEAF* (*CLF*) give early flowering plants with curled leaves. This phenotype is caused by mis-expression of the floral homeotic gene *AGAMOUS* (*AG*) in leaves, so that *ag* mutations largely suppress the clf phenotype. Here, we identify three mutations that suppress *clf* despite maintaining high *AG* expression. We show that the suppressors correspond to mutations in *FPA* and *FT*, two genes promoting flowering, and in *SEPALLATA3* (*SEP3*) which encodes a co-factor for AG protein. The suppression of the clf phenotype is correlated with low *SEP3* expression in all case and reveals that *SEP3* has a role in promoting flowering in addition to its role in controlling floral organ identity. Genetic analysis of *clf ft* mutants indicates that *CLF* promotes flowering by reducing expression of *FLC*, a repressor of flowering. We conclude that *SEP3* is the key target mediating the clf phenotype, and that the antagonistic effects of *CLF* target genes masks a role for *CLF* in promoting flowering.

## Introduction

Plants usually flower at specific times of year, in order to align flowering with periods when pollinators are available and conditions are favourable for growth and fruit set. To achieve this, flowering time is regulated by environmental signals, primarily temperature and photoperiod, and also by intrinsic factors such as the age of a plant. Genetic analysis in Arabidopsis has identified the key components of several flowering pathways, including the photoperiod and vernalization pathways, which mediate responses to daylength and temperature, and the autonomous pathway, which promotes flowering independently of environmental signals [1]. Importantly, the output of these diverse pathways ultimately converges on the control of a few key target genes, termed floral integrators. In addition, genetic analysis suggests that flowering is controlled epigenetically, through factors that act on chromatin of these integrator genes to alter their transcriptional activity. The epigenetic control of flowering is best defined for the vernalization pathway, where long periods of cold such as occur in winter trigger a stable epigenetic change that promotes flowering [2]. In the absence of vernalization, a group of epigenetic repressors termed Polycomb-group (Pc-G) genes play a role in repressing flowering [3], [4]. However, the Pc-G regulate genes with opposite effects on flowering, and the relevance of this has not been clear [5].

In Arabidopsis the photoperiod pathway promotes flowering in response to long days. The output of this pathway involves two integrator genes, *FT* and *SUPPRESSOR OF CONSTANS1* (*SOC1*) both of which promote flowering [6], [7]. *FT* is expressed in vasculature of leaves in long, but not short, days. It encodes a small protein which likely corresponds to florigen, the mobile signal promoting flowering, reviewed recently in [8]. FT protein moves through the phloem from leaves to the shoot apex, where it associates with FD, a HD-ZIP transcription factor, and activates genes such as *LFY*, promoting floral meristem identity [9]. *SOC1* encodes a MADS box transcription factor expressed in the shoot apical meristem and is one of the earliest markers of the floral transition [7], [10]. Genetic analysis suggests that *FT* and *SOC1* act in parallel to promote flowering [7]. The vernalization and the autonomous pathways converge on the activity of *FLC*, which encodes a MADS box transcription factor [11], [12]. FLC is a strong repressor of flowering, largely because it binds *FT* and *SOC1* and represses their expression [10]. *FLC* levels are reduced by the autonomous pathway, which comprises a group of genes with disparate functions. Several members have been found to function in pathways other than flowering. For example, *FCA* and *FPA* both encode RNA binding proteins which regulate mRNA 3′-end processing and poly-adenylation of many genes other than *FLC* [13], [14], [15], [16], [17]. Exactly how *FPA* and *FCA* reduce *FLC* activity has been unclear, as neither seemingly affect processing of the *FLC* transcript. However, they were recently shown to regulate the poly-adenylation site selection of antisense *FLC* transcripts with possible consequences for sense *FLC* transcription rates [14], [15]. In backgrounds with high *FLC* levels, for example autonomous pathway mutants, vernalization treatment is necessary to reduce *FLC* activity and permit flowering. *FLC* levels progressively decline during cold periods [11], [12], and are maintained at low levels when plants are returned to warm conditions. The maintenance of *FLC* repression after vernalization is implemented by Polycomb-group (Pc-G) proteins [2].

The Pc-G are a large group of transcriptional repressors which were first identified from genetic screens in Drosophila, on account of their role in regulating homeotic gene expression. Their protein products associate in several complexes, one of which, Polycomb Repressive Complex 2 (PRC2) is widely conserved between animals and plants [18]. Consistent with a role in the epigenetic control of gene expression, the PRC2 has a biochemical activity towards chromatin, specifically catalysing trimethylation of lysine 27 on histone H3 (H3K27me3) [19], [20], [21], [22]. H3K27me3 is correlated with transcriptional repression and to date the PRC2 is the only enzyme known that produce this mark. The catalytic unit of the PRC2 is a SET domain protein first identified as Enhancer of zeste (E[Z]) in Drosophila and represented by three homologues in Arabidopsis, of which only two - CURLY LEAF (CLF) and SWINGER (SWN) - are expressed after germination [23]. Whereas *swn* mutants are without apparent phenotype, *clf* mutants are small early flowering plants with narrow, upwardly curled leaves. The clf phenotype is largely caused by mis-expression of the floral homeotic gene *AGAMOUS* (*AG*). In wild-type plants, *AG* is only expressed in flowers where it specifies the identity of stamens and carpels in whorls 3 and 4. In *clf* mutants *AG* is expressed outside the flower in vegetative tissues such as leaves and cotyledons. The clf phenotype is largely caused by ectopic *AG* activity as *clf ag* double mutants show near wild type leaf morphology and flowering time [3]. Several other genes, including *APETALA3*, *SEPALLATA3* (*SEP3*), *FT* and *FLC* have also been found to be mis-expressed in clf backgrounds but the relevance of this for Pc-G function has not been clear [5], [24], [25].

We have conducted a genetic screen for modifiers of the clf phenotype and identified suppressors corresponding to *fpa*, *sep3* and *ft* mutant alleles. We show that all three genes are mis-expressed in *clf* mutants and are direct targets of *CLF* as their chromatin is enriched for H3K27me3 the levels of which are strongly depleted in *clf* backgrounds. Genetic analysis indicates that *SEP3* mediates the clf phenotype and that *FT* mis-expression in *clf* mutants masks a role for *CLF* in promoting flowering.

## Materials and Methods

### Plant materials and growth conditions

Plants were grown under LD (16 h light/8 h dark) or SD (8 h light/16 h dark) conditions in controlled environment rooms at 21°C on shelves with fluorescent lighting. Vernalization treatments were performed by sowing seeds on soil, placed in darkness at 4°C for 4 weeks, then transferred to LD or SD conditions at 21°C. The soil used was a mix of Levingtons F2 compost, perlite and sand in proportions 150∶60∶40. The null *clf-50* allele is in Ws background, the *clf-28* (Salk 139371) and *clf-81* alleles are in Col-0 background [26]. The *SEP3::GUS* reporter (Col-0) was provided by Dr Hao Yu (Temasek laborartory, Singapore). All other alleles are in Col-0 background. *ft-10* and *soc1-1* were provided by G. Coupland, *fpa-7* by G. Simpson, *sep3-2* by M. Yanofsky, *flc-3* by R. Amasino.

### T-DNA mutagenesis

The T-DNA mutagenesis was performed in the *clf-50 pCLF::CLF-GR* conditional mutant background in which the clf mutant phenotype is rescued if plants are grown in presence of the steroid dexamethasone [26]. The M_0_ generation was sprayed with 10 µM dexamethasone every 3 days to provide vigorous, fertile plants suitable for floral dip transformation. The M_0_ generation was transformed using Agrobacterium strain GV3101 mp90rk carrying the activation tagging construct pJG41, a derivative of pSKI074 [27]. The pJG41 construct carries an extra selectable marker, *At2S3::GFP*, which renders transgenic seed green fluorescent when viewed under UV illumination [28]. To make pJG41, pSKI074 was cleaved with *Hind*III and made blunt-ended. The plasmid pFP101 (gift of Francois Parcy) was partially digested with *EcoR*I, cut with *Kpn*I and a fragment corresponding to the entire *pAt2S3::GFP* reporter was gel purified, made blunt-ended and ligated to the linearised blunt pSKI 074. The resulting construct confers both kanamycin resistance and seed fluorescence as independent selectable markers. Primary transformants (M_1_ generation) were selected on sterile tissue culture medium containing ½ MS salts (Duchefa), 0.3% sucrose, kanamycin (50 µg/ml), 10 µM dexamethasone and resistant plants were transferred to soil and sprayed with dexamethasone (10 µm) every 3 days. M_2_ seed were collected from bulks of two individuals and M_2_ families were grown on soil without dexamethasone induction and screened for rare families with a suppressed clf phenotype.

### Molecular cloning of sequences flanking T-DNA inserts

DNA flanking the T-DNA right border was obtained using the plasmid rescue technique [27]. To isolate DNA flanking the left border, the genome walker PCR technique was used as previously described [29] with the exception that the pSKI074 specific primers Genewalker LB1 5′-GTTTCTCATCTAAGCCCCCATT and Genewalker LB2 5′-ACGTGAATGTAGACACGTCGAA were used in place of primers LBa1 and LBb1.

### Western blot analysis

For anti-FPA western, antibody and protein extraction method were as described [14], [30]. For detection of AG, antibody and protein extraction were as described [31]. Separation of proteins by SDS PAGE gel electrophoresis, protein transfer to nitrocellulose membranes and protein detection were performed according to standard procedures.

### Gene expression analysis

RNA was extracted from whole seedlings using Qiagen plant RNeasy kits. For first strand cDNA synthesis, 3 µg of total RNA was incubated with 1 µg oligo dT primer (5′-VNTTTTTTTTTTTTTTT) at 65°C for 5 minutes in a 10 µL volume, rapidly cooled on ice, then incubated at 42°C for one hour in a 20 µL reaction containing 1× RT buffer (Promega), 1 ul MMLV reverse transcriptase (Promega), 1 ul RNasin (Promega) and 500 uM dNTP. The reaction was terminated by incubation at 65°C for 15 minutes and the cDNA diluted 1/10 with water. Real time PCR analysis was performed using a Roche LightCycler 480 and 10 µL reactions containing 5 µL diluted cDNA, 1× Sybr Green I mix (Roche) and 200 µM primers. Each 10 µL reaction was triplicated (technical replicates) and for each genotype three biological replicates (i.e. independent plant samples) were made. Primer efficiencies were calibrated using a cDNA dilution series and Cp values and relative amounts were determined using the 2^nd^ derivative max method in the Lightcycler 480 software (Roche). Results from different samples were normalised relative to expression of the *EiF4A* gene. Primers were as follows: *EIF4A* 5′-TTCGCTCTTCTCTTTGCTCTC and 5′-GAACTCATCTTGTCCCTCAAGTA; *AG* 5′-TCCGAGTATAAGTCTAATGCC and 5′-GCCTATATTACACTAACTGGAGAG; *SEP3* 5′-TATGACGCCTTACAGAGAACC and 5′-ATACCCATCAGCTAACCTTAGTC; *SEP1* 5′-TCAACAACAAACCTGCCAAA and 5′-ATGTAACCGTTTCCCTGCTG; *SEP2* 5′-TGGCTCCATTGAAGTCAACA and 5′-CTGAGCACACTGGATGGCTA; *SEP4* 5′-TTTCTCTAACCGTGGCAAGC and TTCCTGAATTGGAGGGTTTG; *FLC* 5′-CGGTCTCATCGAGAAAGCTC and 5′-CCACAAGCTTGCTATCCACA; *FT* 5′-CCTCAGGAACTTCTATACTTTGGTTATGG and 5′-CTGTTTGCCTGCCAAGCTGTC.

### Chromatin immunoprecipitation (ChIP)

Seedlings were grown for 12 days in sterile tissue culture on MS medium, roots were cut away and the remaining shoots harvested for assay. ChIP assays were performed as in Finnegan et al [32]. Antibodies recognising H3K27me3 (07-449) and H3K4me3 (07-473) were from Millipore. Assays were performed on two independent biological samples with similar results. The relative amounts of DNA in the input, no antibody control and IP samples were quantified by real time PCR using a Roche LightCycler 480 (Roche) as described above, with three technical replicates for each sample. Enrichment was quantified as the proportion of the input DNA that was recovered in the IP sample. The primers used were as follows: *AG* 5′-CCCAAAGATTTTAGTGCCTCA and 5′-GGTTCAAGAGGGCAATCAC; *FLC* 5′-GAGGCACCAAAGAAACAAGG and 5′-TCGCCCTTAATCTTATCATCG; *SEP3-M* 5′-CTTTTGATTCTGGGGGTCGT and 5′-GATGAATCCCATCCCCAAGT; *SEP3-2* 5′-GTGTTGGTGAGAGTGGAACTC and 5′-ACTCTCAGACTCAACTATATACCC; *FT* 5′-GTGGCTACCAAGTGGGAGAT and 5′-TAACTCGGGTCGGTGAAATC; *FUS3* 5-CGTGGGAAATAGGAGGCATA and 5′-GTGGCAAGTGTTGATCATGG.

### Histochemistry

To assay activity of the *GUS* reporter gene, whole seedlings were stained, cleared and photographed as described in Chanvivattana et al [23].

## Results

### A genetic screen for modifiers of the clf mutant phenotype

Because there is considerable redundancy between the closely related Pc-G genes *CLF* and *SWN* [23] even null *clf* mutants have an intermediate level of Pc-G activity; consequently, we reasoned that the clf phenotype would be sensitised to small changes in activity of *CLF* target genes, for example due to mutations in the target genes themselves or in genes that regulate their activity such as trx-G or Pc-G members. We therefore mutagenised the null *clf-50* mutant background, using random T DNA integration, and screened the M_2_ generation for mutations suppressing the clf phenotype (see materials and methods). Here, we describe three strong suppressor mutations, which gave near wild-type plants, and define targets that mediate the clf phenotype. A second category of mutants, affecting other genes involved in chromatin modification, will be described elsewhere.

### *fpa* mutations suppress *clf* by increasing *FLC* activity

**Figure 1:**
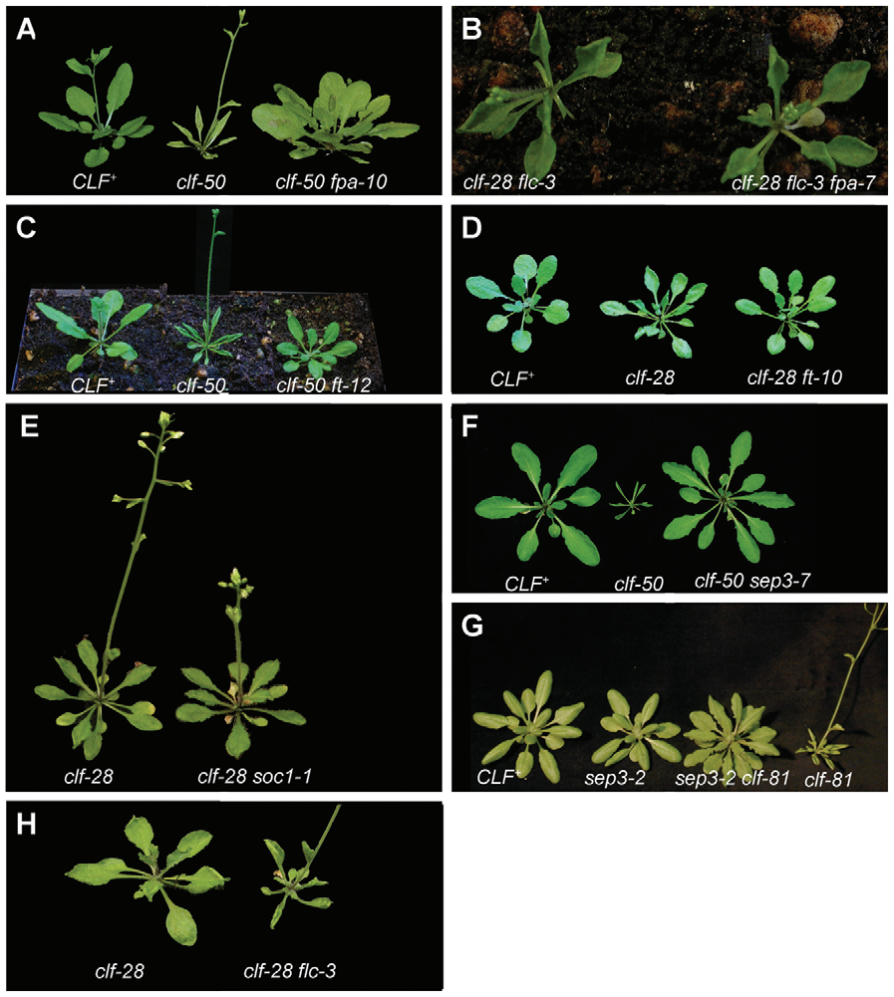
Mutants suppressing the clf phenotype. Rosettes of four week old plants that were grown in long day [except (**G**), short day grown plants], at which time the early flowering and leaf curling of clf mutants is easily seen. (**A**) The *fpa* mutation suppresses the leaf curling and early flowering of *clf-50* and results in late flowering. (**B**) *the clf-28 flc-3 fpa-7* triple mutant *resembles clf-28 flc-3* mutants and shows early flowering and leaf curling. *FLC* activity is therefore required for the suppression of *clf* by *fpa*. (**C, D**) *ft* mutations also suppress *clf* mutations and cause late flowering. (**E**) *soc1* mutations do not suppress the early flowering and mild leaf curling of *clf-28* mutants. (F, G) *sep3* mutations suppress the *clf* phenotype. (**H**) the *flc-3* mutation enhances the leaf curling, small size and early flowering of *clf-28* mutants.

**Figure 2:**
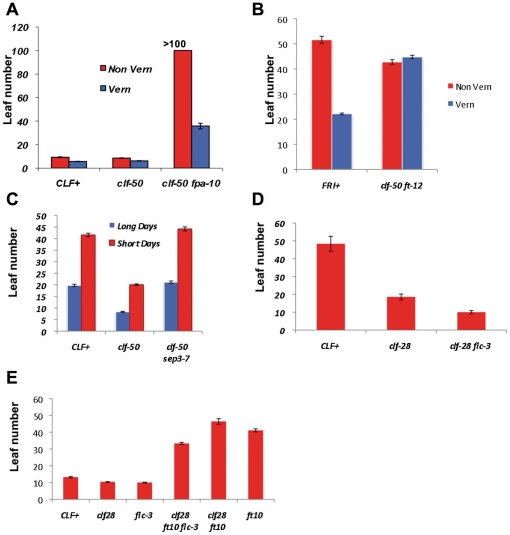
Effects of suppressor mutants upon flowering time. Flowering time was recorded as the number of rosette leaves at bolting, thus late flowering plants have more rosette leaves. Plants were grown in long days unless otherwise stated. Error bars show standard error of mean calculated from at least 10 plants. (**A**) The *clf-50 fpa-10* mutant shows a strong vernalization reponse. (**B**) The *clf-50 ft-12* mutant does not respond to vernalization treatment. (**C**) The *clf-50 sep3-7* mutant flowers at similar time to wild type, thus *SEP3* ^+^ activity is needed for early flowering in the *clf* background. (**D**) The *flc-3* mutation enhances the early flowering of *clf-28* mutants, revealing that *FLC* activity delays flowering in the *clf* background. Plants grown in short days, where the effects of *clf* on flowering time are most obvious (**E**) *clf-28 ft-10* mutants flower later than *ft-10* mutants due to *FLC* ^+^ activity.

**Figure 3:**
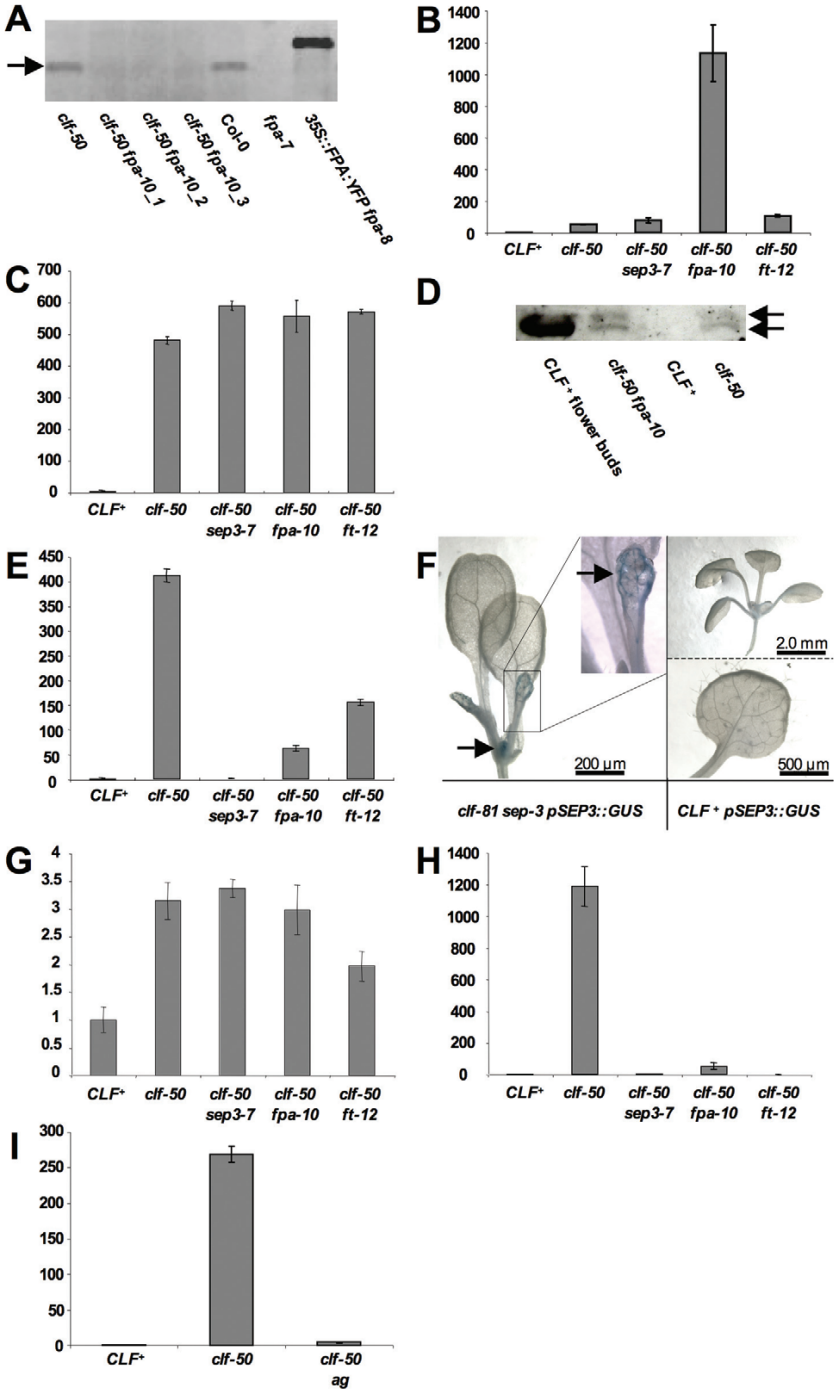
Gene expression in suppressor mutants. (**A**) Western blot analysis of FPA protein levels. Three independent *clf-50 fpa-10* samples were processed. Note that no protein is detected in the null *fpa-7* control, whereas in extracts from a *35S::FPA-YPA* transgenic line a larger product corresponding to the *FPA-YFP* fusion protein is detected, confirming the specificity of the antibody for FPA. No FPA protein is detectable in *fpa-10* extracts, indicating that *fpa-10* is likely a null allele. (**B**) Real time PCR analysis of *FLC* expression. (**C**) Real time PCR analysis of *AG* expression, showing high *AG* expression in suppressor mutants. (**D**) Western blot analysis of AG protein expression. The AG antibody detects two proteins of about 29 kDa that are specific for AG, the smaller band possibly representing a truncated product or spurious translation initiation event (Riechmann et al., 1999). AG protein is strongly detected in wild type flowers but not in leaves. Weak expression is found in *clf-50* and *clf-50 fpa-10* leaves. (**E**) Real time PCR analysis of *SEP3* expression. (**F**) Histochemical staining of GUS reporter gene activity. *SEP3::GUS* is not expressed in wild type leaves but shows weak expression in vasculature of *clf-81* leaves (enlarged in inset). (**G**) Real time PCR analysis of *SEP2* expression. (**H**) Real time PCR analysis of *FT* expression (**I**) Real time PCR analysis of *SEP3* expression. Error bars in real time PCR experiments represent standard error of mean of three independent samples (biological replicates). Expression was normalised relative to the *EiF4A* gene, and is expressed relative to expression in wild type. In **B**, **C**, **E**, **G**, **H** whole seedlings less roots of 20 day old short day grown seedlings were used. In **I** rosette leaves of long day plants at 21 days were used.

We identified a mutant which strongly suppressed the leaf curling and early flowering of *clf-50* mutants. The double mutant with *clf-50* was also very late flowering relative to the *CLF^+^* (Ws) background (Fig. 1A and Fig. 2A). Late flowering mutants can be further characterised by their response to vernalization treatments, which restore normal flowering time to mutants in the autonomous flowering pathway flower but not those in the photoperiodic pathway [33]. The suppressor mutant showed a strong response to vernalization treatments (Fig. 2A), suggesting that it affected a gene in the autonomous pathway. Consistent with this, molecular cloning (see materials and methods) revealed that the mutant harboured a T-DNA insertion in the first intron of *FPA* (see Fig. S1A in supplementary material), a gene acting in the autonomous pathway [34]. The novel *fpa* allele, designated *fpa-10*, is likely a null allele as western analysis using an anti-FPA antibody showed that FPA protein, readily detected in wild-type and *clf-50* plants, is absent from *clf-50 fpa-10* (Fig. 3A). The *FPA* gene promotes flowering by decreasing expression of *FLC*, a repressor of flowering [35]. Consistent with this, the *clf-50 fpa-10* mutant had greatly elevated *FLC* levels relative to wild-type and *clf-50* plants (Fig. 3B). Although the effects of *fpa* mutations on flowering time are solely due to increased *FLC* expression [35], *FPA* is known to regulate many genes other than *FLC* [13], [14]. To test whether the suppression of *clf* by *fpa* mutants is solely due to increased *FLC* activity or rather involves other *FPA* targets, we made *clf-28 fpa-7 flc-3* triple mutants. This abolished the suppression, i.e the triple mutants had narrow curled leaves like those of *clf-28 flc-3* mutants, suggesting that the suppression of *clf* by *fpa* is mediated solely by high *FLC* levels (Fig. 1B). Lastly, we obtained an additional late flowering suppressor mutant and found that this harboured a T-DNA insertion in the *FCA* gene, another member of the autonomous flower promoting pathway (Fig. S2 in supplementary data). Together these results indicate that autonomous pathway mutants suppress clf by causing elevated *FLC* activity.

The clf phenotype is known to be caused by ectopic expression of *AG* in leaves of *clf* mutants [3]. To test whether *fpa* mutants suppressed the clf phenotype by reducing *AG* activity, we first measured levels of *AG* RNA in *clf-50 fpa-10* mutants. Unexpectedly, *AG* mRNA was expressed as strongly in *clf-50 fpa-10* as in *clf-50* mutants, despite the lack of leaf curling (Fig. 3C). The *FPA* gene acts by controlling the location within the mRNA of its targets where cleavage and polyadenylation occurs, often leading to changes in the protein product encoded [14]. To test whether the AG protein product was affected by *fpa* mutation, we analysed protein levels on western blots using a previously isolated antibody to AG protein [31]. We detected two protein products of about 29 Kda that were specific to *AG^+^* plants, and these were expressed at a similar level in *clf-50* and *clf-50 fpa-10* backgrounds (Fig. 3D). We concluded that the suppression of *clf* by *fpa* mutation occurs independently of *AG*, and that *CLF* therefore must have other target genes that are relevant for its mutant phenotype.

### *ft* mutations suppress *clf*

We obtained another mutation which suppressed *clf-50* and caused late flowering relative to the *clf-50* and *CLF+* (Ws) backgrounds (Fig. 1C), suggesting that it also affected a gene promoting flowering. Vernalization treatments had little effect on flowering time of this mutant, suggesting it might affect a gene in the photoperiodic rather than the autonomous flower promoting pathway (Fig. 2B). Consistent with this, molecular cloning revealed that the mutant harboured a T-DNA insertion in the first intron of the *FT* gene (see Fig. S1B in supplementary material) and thus corresponded to a novel *ft* allele, designated *ft-12*. When the *clf-50 ft-12* mutant was back-crossed to wild-type (Ws) the resulting F_1_ plants had normal flowering time (13 of 13 plants), indicating that *ft-12* was a recessive loss of function mutation. The late flowering phenotype co-segregated with the T-DNA insertion, as all late flowering plants (29 of 144 F_2_ plants) identified in F2 populations from crosses to *CLF^+^* (Ws) were homozygous for a selectable marker (seed fluorescence) carried by the T DNA. To confirm that *ft* mutations can suppress the *clf* phenotype, we made an independent *clf ft* double mutant that combines the null *clf-28* and *ft-10* alleles in the Col-0 background. The *clf-28 ft-10* double mutant suppressed the early flowering and leaf curling of the *clf-28* mutation, confirming that *FT* activity is required for the *clf* phenotype (Fig. 1D). *FT*, together with the *SOC1* gene, is known to integrate the outputs from the different pathways promoting flowering in *Arabidopsis* [6], [7]. The *SOC1* gene carries H3K27me3 methylation [36] and is therefore likely to be a Pc-G target. To test whether *SOC1* activity was also required for the clf phenotype, we made *clf-28 soc1-1* double mutants. However, the double mutants had similar leaf morphology and early flowering as *clf* single mutants (Fig. 1E). Thus *FT* but not *SOC1* activity was necessary for the clf phenotype. To test whether the suppression of *clf* by *ft* mutation was caused by reduced *AG* activity, we measured *AG* RNA levels in *clf-50 ft-12* double mutants. However, *AG* was expressed as strongly in *clf-50 ft-12* doubles as it was in *clf-50* plants (Fig. 3C), indicating that the suppression was not mediated by *AG*.

### *sep3* mutations suppress the leaf curling and early flowering of *clf* mutations

We identified a third suppressor mutation (Fig. 1F) which, unlike the previous two mutations, had little effect on flowering time. When the mutant was backcrossed to the *clf-50*, the resulting F_1_ plants all had a *clf* phenotype and the F_2_ generation segregated about ¼ for the suppressed phenotype (15 in 73 plants), consistent with the suppression being caused by a single recessive mutation. The mutant plants harboured a T-DNA insertion that disrupted both the *SEPALLATA3* (*SEP3*) gene and an adjacent gene of unknown function (*At1g24265*, see Fig. S1C in the supplementary material). We genotyped ten plants from the segregating F2 and found that the novel *sep3* mutation, designated *sep3-7*, co-segregated with the suppressed phenotype (data not shown). To confirm that disruption of *SEP3*, rather than the neighbouring *At1g24265* gene, suppressed the clf phenotype we created a second *sep clf* double mutant using the independent *sep3-2* and *clf-81* alleles in the Col-0 genetic background. Again, a strong suppression resulted (Fig. 1G). Lastly, we recently obtained an additional suppressor mutant with similar phenotype to *sep3-7* and found that it contained an T-DNA insertion in the penultimate exon of *SEP3* and therefore constituted an independent *sep3* allele designated *sep3-8* (data not shown). Together these results show that *SEP3* activity is required for the clf phenotype.

To test whether *sep3* mutations also suppress the early flowering of *clf* mutants, we measured flowering times in long and short days. The *clf-50 sep3-7* plants flowered at the same time as wild-type (Ws) plants in long days and slightly later in short days (Fig. 2C, 41.6±0.78 leaves in Ws versus 44.2±0.93 in *clf-50 sep3* see Fig. 2C). Thus *SEP3* activity is needed for the early flowering of *clf* mutants.

To test whether *SEP3* might normally have a role in promoting flowering, i.e. in wild-type backgrounds as well as in *clf* mutant backgrounds, we crossed the *sep3-7 clf-50* suppressor mutant to the wild-type Ws progenitor background and screened the flowering time of the resulting F2 in short and long days. About 3/16 of the resulting F2 plants are predicted to be *CLF^+^ sep3-7* genotype, but we did not observe significant differences in flowering time other than some early flowering plants with curled leaves that presumably were *clf-50 SEP^+^* (data not shown). This suggests that *SEP3* activity is not needed for normal flowering time.

*SEP3* is one of four closely related genes (*SEP1-4*) that act redundantly and encode co-factors for the activity of AG and other floral homeotic proteins in flowers [37], [38]. The suppression of *AG*-induced leaf curling in *clf* mutants by *sep3* mutations suggests that *SEP3* is also needed for *AG* activity in leaves, but has less redundancy with the other *SEP* genes in leaves. We therefore measured the expression of the *SEP* genes in wild-type and mutant seedlings (Fig. 3E). *SEP3* expression was indeed strongly upregulated in *clf-50* seedlings relative to wild-type (about 400 fold). In addition, when we introduced a *SEP3::GUS* reporter gene fusion [39] into the *clf-50* mutant background, we observed GUS activity in leaves of *clf-50* but not wild-type plants (Fig. 3F). By contrast, *SEP2* expression showed a slight (three fold) increase in expression in *clf-50* mutants (Fig. 3G), and expression of *SEP1* and *SEP4* was not detectable in wild-type or mutant seedlings (not shown). Together, these results indicated that only *SEP3* is strongly misexpressed in *clf* leaves, so it has less redundancy with the other *SEP* genes than in flowers, where all four genes are expressed.

Previous studies have shown that the *SEP3* gene is required for *AG* activity in two ways. Firstly, the SEP3 protein is a co-factor needed for AG protein activity [37], [40]. Secondly, SEP3 protein can activate *AG* transcription in flowers [39], [41]. To test whether *SEP3* has a role in activating *AG* expression in *clf* mutants, we quantified *AG* mRNA levels in seedlings. We found that *AG* was strongly mis-expressed in *clf* mutants regardless of *SEP3* activity (Fig. 3C). In addition, western blot analysis using an anti-AG antibody indicated that AG protein is present at similar levels in *clf* and *clf sep3* leaves (Fig. 3D). These results suggest that *SEP3* is needed for the activity of the AG protein, but not for its stability or for *AG* transcription in *clf* mutants.

To test whether *AG* activity was needed for expression of *SEP3* in *clf* mutants, we measured *SEP3* expression in leaves of wild-type, *clf-50* and *clf-50 ag* mutants. *SEP3* expression was strongly reduced in *clf-50 ag* leaves (Fig. 3I). Thus, although *AG* transcription in *clf* leaves is independent of *SEP3*, *SEP3* transcription requires *AG*.

### Antagonistic interactions between *CLF* target genes

**Figure 4:**
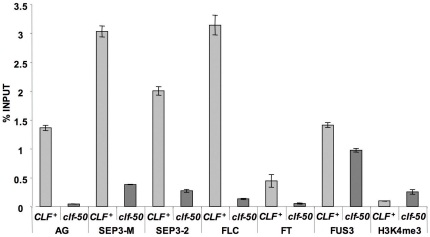
Effect of the *clf* mutation on histone methylation. ChIP analysis using 12 day old seedlings. Results show H3K27me3 levels at different genes, other than the rightmost bars which show H3K4me3 levels at the *AG* gene. The SEP3-2 primers amplify a region of the *SEP3* promoter previously implicated in regulation of *SEP3* by Pc-G proteins [63], SEP3-M amplify part of the large first intron of *SEP3*. Error bars are standard error of mean of three technical replicates. The experiment was repeated on independent samples with very similar results as shown in supplementary data Figure S3.

Our genetic analysis indicated that in addition to *AG*, the *FT*, *SEP3* and *FLC* genes are relevant for the clf phenotype. It is likely that they are direct targets of the Pc-G. All three genes are mis-expressed in *clf* mutant seedlings (Fig. 3B, E, H). In addition, all three are marked with H3K27me3 methylation, which is characteristic of Pc-G targets [36]. To test whether *CLF* is required for normal H3K27me3 levels at these genes, we performed ChIP assays using wild-type and *clf-50* mutant seedlings. As expected, all three genes were strongly enriched with H3K27me3 relative to a control gene that is not a Pc-G target. In addition, all three genes had reduced H3K27me3 methylation in *clf* mutants (Fig. 4 and Fig. S3 in supplementary data), consistent with their mis-expression in clf. By contrast, the *FUSCA3* (*FUS3*) gene, a Pc-G target which is mis-expressed in *clf swn* mutants but not *clf* mutants [42], showed less reduction in H3K27me3 in *clf* mutants (Fig. 4). To ensure that the reduced H3K27me3 in *clf* mutants did not simply reflect poor quality extracts from the mutants, we immunoprecipitated the same chromatin extracts using an antibody against the active chromatin mark H3K4me3, and in this case saw increased methylation in the *clf* mutants (Fig. 4).

The *CLF* targets have antagonistic effects on flowering, as *FT* and *SEP3* promote early flowering in *clf* mutants, whereas *FLC* represses flowering. This suggested that the clf phenotype may represent a balance between these opposing activities. To test this, we first removed *FLC* activity in a *clf* background by combining the null *flc-3* mutation and *clf-28* mutations in a uniform Col-0 background. The double mutants showed stronger leaf curling (Fig. 1H) and much earlier flowering than *clf-28* single mutants (Fig. 2D), indicating that *FLC* indeed antagonises the effects of *FT/SEP3/AG* on flowering in *clf* mutants. Secondly, we measured the effects on flowering time of removing *FT* and *FLC* activity in the *clf* background. Strikingly, *clf-28 ft-10* mutants were later flowering than *ft-10* mutants, despite the fact that *clf-28* single mutants are early flowering. By contrast, *clf-28 ft-10 flc-3* triple mutants flowered earlier than *ft-10* mutants (Fig. 2E). Together, these results show that the effects of elevated *FLC* expression in *clf* mutants are masked by increased *FT* activity – in the absence of the early flowering conferred by *FT*, increased *FLC* activity makes *clf* mutants late flowering.

### Mutual activation of *SEP3* and *FT* in *clf* mutants

Increased expression of *FT* activates *SEP3* in leaves [43] suggesting that the increased *SEP3* activity in *clf* mutants might be due to the increased *FT* expression. Consistent with this, *SEP3* expression was much lower in *clf-50 ft-12* mutants than it was in *clf-50* mutants, although it was still higher (about 150 fold) than in wild-type (Fig. 3E). By contrast, activation of *AG* in *clf-50* was independent of *FT* activity (Fig. 3C). We also found that *FT* misexpression was considerably reduced in *clf-50 sep3-7* relative to *clf-50* and barely higher (about four fold) than in wild-type (Fig. 3H). Thus, *SEP3* is required for the activation of *FT* expression that causes early flowering in *clf* mutants.

An unexpected feature of the suppressor mutants was that although they largely eliminated leaf curling, they showed no reduction in *AG* activity. However, in all cases *FT* and *SEP3* expression was strongly reduced relative to *clf-50*, although still slightly higher than in wild-type. This suggested that the suppression of leaf curling is caused by reduced *SEP3* and *FT* expression rather than *AG*. It is likely that *FT* activity is required in addition to *SEP3* and *AG*, because in *clf-50 ft-12* mutants *SEP3* activity is still relatively high compared to wild-type (about 150 fold increased, Fig. 3E) yet leaf curling is suppressed.

## Discussion

Whole genome profiling of H3K27me3 suggests that the Pc-G may bind to many thousands of targets in Arabidopsis [36], [44]. However, the biological relevance of this binding is uncertain, as a relatively low proportion of the targets are mis-expressed in Pc-G mutants [45]. Previous work showed that *AG* is necessary for the clf phenotype and that mis-expression of *AG* causes leaf curling [3], [46]. Here, we identify additional mutants that strongly suppress the clf phenotype. Strikingly, the mutants retain strong *AG* expression in leaves. Our analysis shows that the *SEP3 FT* and *FLC* genes are also key for the clf phenotype. In particular, we confirm a role for *SEP3* in promoting flowering, consistent with a previous study showing that SEP3 over-expression causes early flowering [47]. These targets have antagonistic effects on flowering and genetic analysis confirms that the clf phenotype represents a balance of factors promoting and delaying flowering.

### High *FLC* levels suppress *clf*

Mutations in *FPA*, which acts in the autonomous pathway promoting flowering, suppress *clf*. Although *FPA* is known to regulate genes other than *FLC* [13], [14] our genetic analysis showed that the suppression is caused by the elevated levels of *FLC* in *fpa* mutants. Thus, when *FLC* is inactivated, *fpa* mutations no longer suppress clf. Despite the suppressed phenotype of *clf fpa* mutants, they express *AG* RNA and protein as strongly as do *clf* mutants. Instead, their levels of *FT* and *SEP3* are strongly decreased. These results are consistent with recent whole genome profiling of sites bound by FLC protein, which showed that *SEP3* and *FT* but not *AG* are targets [48]. Because the SEP proteins are required for the activity of AG and other floral homeotic proteins [37], [38], it is the decrease in *SEP3* that is likely most important for suppression of leaf curling in *clf* backgrounds. *SEP3* levels in *clf fpa* are still higher than in wild-type, which suggests either that there is a threshold of *SEP3* activity required for leaf curling and early flowering or that *FT* activity is also necessary for leaf curling. Consistent with the former, *35S::SEP3* transgenes give variable effects on leaf curling, presumably relating to expression levels [37].

Our results and those of other groups show that *CLF* represses *FLC*, so that in *clf* mutants *FLC* expression is increased [5], [49], albeit much less so than in *fpa* or *fca* mutant backgrounds. The relatively minor effects of *clf* mutation on *FLC* activity may reflect redundancy between *CLF* and *SWN*. Indeed, microarray analysis (data at http://affy.arabidopsis.info/narrays/experimentpage.pl?experimentid=425) shows that *clf swn* seedlings show much higher increases in *FLC* expression compared to wild type (118 fold) than do *clf* mutants (8 fold). It is striking that in the absence of *FPA* (or *FCA*) activity, *CLF* is unable to repress *FLC*. One possibility is that *FPA* and *FCA* are needed for CLF to be recruited to or act on *FLC*. Previous studies have shown that *FPA* and *FCA* are needed for recruitment of FLD, a H3K4me2 histone demethylase, to *FLC* [50]. It is possible that the removal of H3K4me2 by FLD is necessary in order for CLF to catalyse H3K27me3 at *FLC*, for example if H3K4me2 inhibits the H3K27me3 methyltransferase. A recent study shows that H3K4me3 inhibits the activity of a reconstituted CLF/EMF2/FIE/MSI1 complex in in vitro assays, and it is plausible that H3K4me2 has a similar effect [51]. There is also a role for *COLDAIR*, a non coding RNA produced from *FLC*, in recruiting *CLF* to *FLC* [52]. *COLDAIR* is expressed most strongly during cold treatments, but knock down experiments suggested that it also has a role in recruiting CLF and repressing *FLC* in the absence of cold treatment [52]. It seems unlikely that *FPA* and *FCA* regulate *COLDAIR* directly via poly-A site selection, as *COLDAIR* apparently lacks a polyA tail at its 3′-end [52], but might act indirectly via their effects on *COOLAIR*, the *FLC* antisense transcript [53].

### Activation of *SEP3* and *AG* in *clf* mutants

In flowers, the four *SEP* genes largely act redundantly as triple and quadruple knockouts are needed to reveal their function [38], [54]. *SEP3* has some discrete functions as *sep3* single mutants have very subtle effects on petal development [47]; in addition, SEP3 protein shows stronger transcriptional activation activity than the other SEP proteins when assayed in onion cells [37]. In *clf* mutant leaves, *SEP3* is absolutely required for curling, so here there is little redundancy with the other *SEP* genes. This probably reflects their expression, as (unlike *SEP3*) *SEP1 SEP2* and *SEP4* showed little activation in *clf*. This raises the question of what activates *SEP3* in *clf* mutants. One factor is *FT*: in *35S::FT* plants, *SEP3* is expressed in leaves [43], and high levels of *SEP3* expression in *clf* mutants is dependent of *FT* activity as in *clf ft* mutants expression of *SEP3* is strongly down-regulated. *AG* activity is also required as in *clf ag* mutants *SEP3* levels are strongly reduced. It is likely that the role of AG is to form an AG/SEP3 complex which autoactivates and stabilises *SEP3* expression. This is consistent with microarray analysis of flower development, where transient induction of *AG* can lead to persistent *SEP3* and *AG* activity via autoregulatory loops in which SEP3/AG complexes bind and upregulate *AG* and *SEP3* [41] Interestingly, the activation of *AG* is independent of *SEP3* in *clf* mutant backgrounds. Thus, *cfl fpa*, *clf ft* and *clf sep3* show high *AG* activity despite low *SEP3* levels. This also shows that unlike *SEP3*, *AG* does not require *FT* for its activation in *clf* leaves.

### Role of *SEP3* in promoting flowering

Our results show that *FT* is needed to activate *SEP3* in *clf* leaves, consistent with a previous study showing that over-expression of *FT* in leaves is sufficient to induce *SEP3* expression [43]. Unexpectedly, we also find that *SEP3* is required for activation of *FT* expression in *clf* mutants. Thus, *clf sep3* mutants have low *FT* levels and flower slightly later than wild-type in short days. *SEP3* is therefore needed to promote flowering via *FT* in *clf* mutants. This raises the question of whether *SEP3* might have any role in promoting the floral transition in wild-type (*CLF^+^* backgrounds) as well. Precocious expression of *SEP3* in leaves using a *35S::SEP3* transgene is sufficient to cause early flowering [37]. In addition, *35S::SEP3-EAR* transgenes (which express a fusion of SEP3 to the EAR transcriptional repression domain and presumably inactivate SEP gene targets), confer late flowering in Arabidopsis [55]. However, it is unlikely that *SEP3* normally promotes flowering in Arabidopsis: firstly, *sep3* mutants showed normal flowering time as in this study and [43]; secondly, *SEP3* expression is not detectable in wild type rosette leaves until after the floral transition [43]. However, *SEP3* may be important in promoting flowering in other species. Thus knockdown of the rice *SEP3* homologue delays flowering [56].

### Antagonism between targets masks Pc-G role in promoting flowering

The targets of *CLF* have opposite roles, either promoting (*FT, AG, SEP3*) or repressing (*FLC*) flowering and leaf curling. The clf phenotype is therefore a balance of these antagonistic factors. Although Pc-G genes are generally thought to repress flowering, as mutants such as *clf* and *emf2* are very early flowering, they also promote flowering as is revealed by the fact that *clf ft* mutants flower later than *ft* mutants. Such antagonism between targets provides one explanation as to why relatively few predicted targets are mis-expressed in Arabidopsis PcG mutants [45], as targets that are repressors may mask the activation of other targets. Similarly, in Drosophila, the activation of several homeobox target genes in Pc-G mutant wing cells prevents the activation of another target, *Distal-less (Dll)* so that effects on *Dll* expression are only visible in mutant backgrounds lacking activity of both the Pc-G and the antagonistic homeobox genes [57].

It is also clear that for Pc-G targets such as *FT*, repression is rapidly overcome during floral induction, for example if short day grown plants are shifted to long days or if the upstream regulator *CONSTANS* (*CO*) is induced using a steroid dependent *35S::CO-GR* transgene, *FT* is activated within one day or two hours, respectively [58], [59]. Similarly, repression of *SEP3* by *CLF* is overcome in *35S::FT* transgenic plants that overexpress *FT*, although normal expression levels of *FT* in long day grown plants are insufficient to overcome Pc-G mediated repression in leaves, at least until late in development [43]. In several other cases it has also been shown that Pc-G mediated repression in plants is relatively easily overcome and mainly affects the dynamics of gene expression rather than providing an insurmountable block [32], [60], [61]. Alternatively, *FT SEP3 FLC*, and *AG*, which are normally activated during adult plant development may differ from other PcG targets (e.g *FUS3*), which are permanently repressed after seed maturation, in Pc-G dependent chromatin modifications other than H3K27me3 [62].

## Supporting Information

Figure S1 **Molecular structure of suppressor mutants.** We isolated the DNA flanking the T DNA insertion causing the suppressor mutation using plasmid rescue and genome walker procedures (see methods). The structures shown are the most straightforward interpretation of the data but more complex arrangement are possible, for example tandem T-DNA insertions. Exons are shown as light blue boxes, start of transcription indicated with an arrow. (**A**) *fpa-10* allele. Recovery by plasmid rescue of *an Eco*RI fragment containing the T DNA right border indicated that the T-DNA insertion was located in the first intron of *FPA*. (**B**) An *Eco*RI fragment containing the T-DNA left border and plant flanking sequences was recovered by the genome walker procedure. Sequence analysis revealed that the T DNA is inserted in the *FT* first intron. (**C**) *sep3-7* allele. Recovery of an *Eco*RI fragment by plasmid rescue indicated that the T DNA insertion at *At1g24265* is associated with a deletion in the neighbouring *SEP3* gene. PCR analysis of genomic DNA confirmed that independent *sep3-7* mutants carried a deletion within this region of the *SEP3* locus (not shown).
(TIF)

Figure S2 **Molecular structure of** ***fca-8902*** **allele.** Exons are shown as light blue boxes, start of transcription indicated with an arrow. (**A**) *fca-8902* allele. We recovered a *Vsp*I fragment and a *Hin*dIII fragment both containing T-DNA left border and plant flanking sequences. Sequence analysis of these fragments suggests a tandem insertion of at least two T DNAs in inverse orientation within the eighth intron of the *FCA* gene. The *FCA* gene produces several transcripts, the gene structure for the beta (functional) transcript is shown (**B**) Suppression of the early flowering and leaf curling phenotype of *clf-50* by *fca* mutation. Long day plants 21 days after germination (dag). (**C**) 9 week old plants grown in long days, showing the late flowering phenotype of *clf-50 fca-8902* double mutants.
(TIF)

Figure S3 **Effect of the** ***clf*** **mutation on histone methylation.** ChIP analysis using 12 day old seedlings. Results show H3K27me3 levels at different genes, experiment was performed on independent samples from those in Figure 4. Error bars are standard error of mean of three technical replicates.
(TIF)
