# Supplementary figures and images for: Antagonistic Roles of SEPALLATA3, FT and FLC Genes as Targets of the Polycomb Group Gene CURLY LEAF
Source: PLoS One. 2012 Feb 17;7(2):e30715. doi: 10.1371/journal.pone.0030715 (PMC3281876; doi:10.1371/journal.pone.0030715)

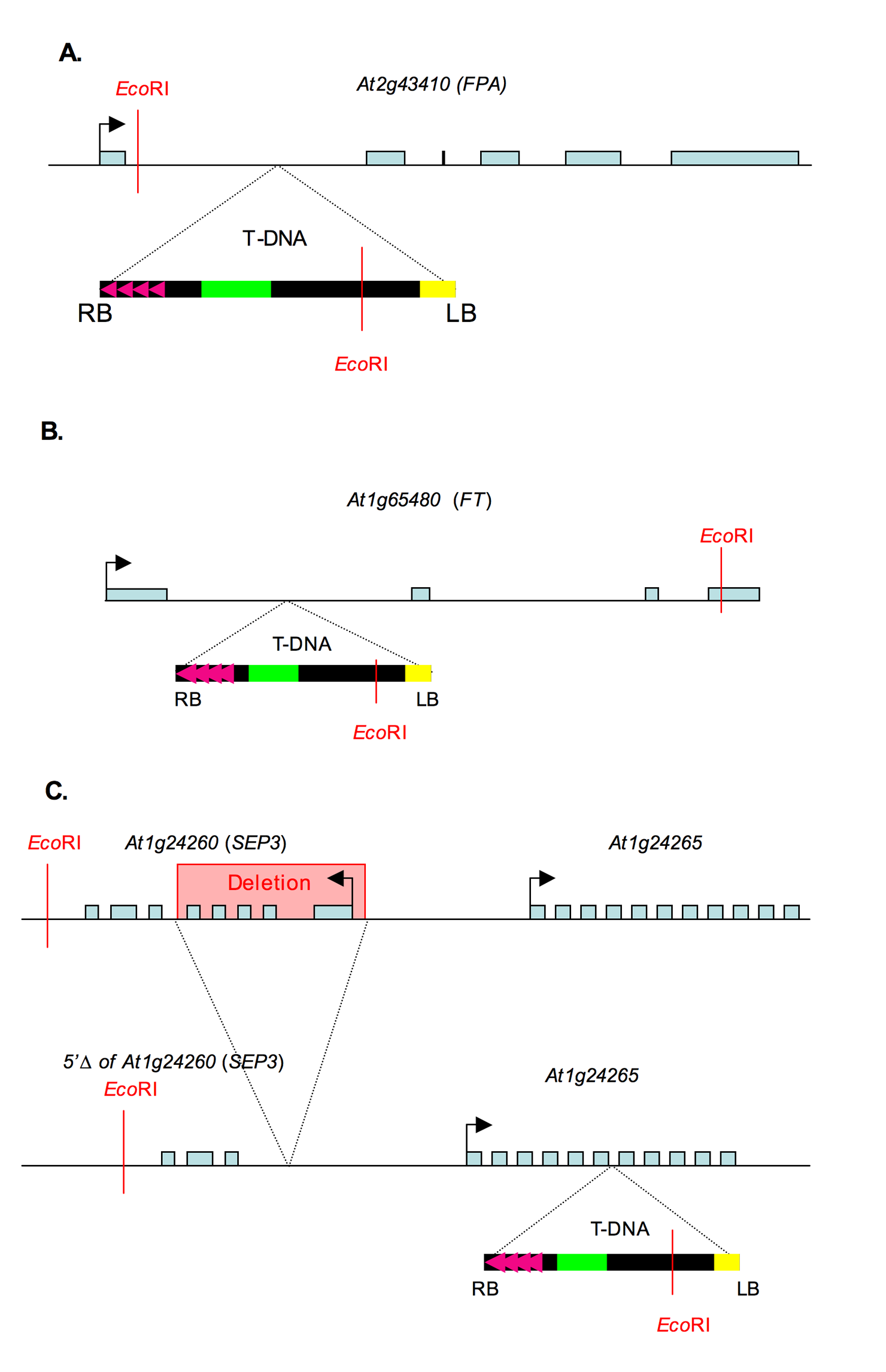

Supplement: Figure S1 — Molecular structure of suppressor mutants. We isolated the DNA flanking the T DNA insertion causing the suppressor mutation using plasmid rescue and genome walker procedures (see methods). The structures shown are the most straightforward interpretation of the data but more complex arrangement are possible, for example tandem T-DNA insertions. Exons are shown as light blue boxes, start of transcription indicated with an arrow. (A) fpa-10 allele. Recovery by plasmid rescue of an EcoRI fragment containing the T DNA right border indicated that the T-DNA insertion was located in the first intron of FPA. (B) An EcoRI fragment containing the T-DNA left border and plant flanking sequences was recovered by the genome walker procedure. Sequence analysis revealed that the T DNA is inserted in the FT first intron. (C) sep3-7 allele. Recovery of an EcoRI fragment by plasmid rescue indicated that the T DNA insertion at At1g24265 is associated with a deletion in the neighbouring SEP3 gene. PCR analysis of genomic DNA confirmed that independent sep3-7 mutants carried a deletion within this region of the SEP3 locus (not shown). (TIF) [file pone.0030715.s001.tif]

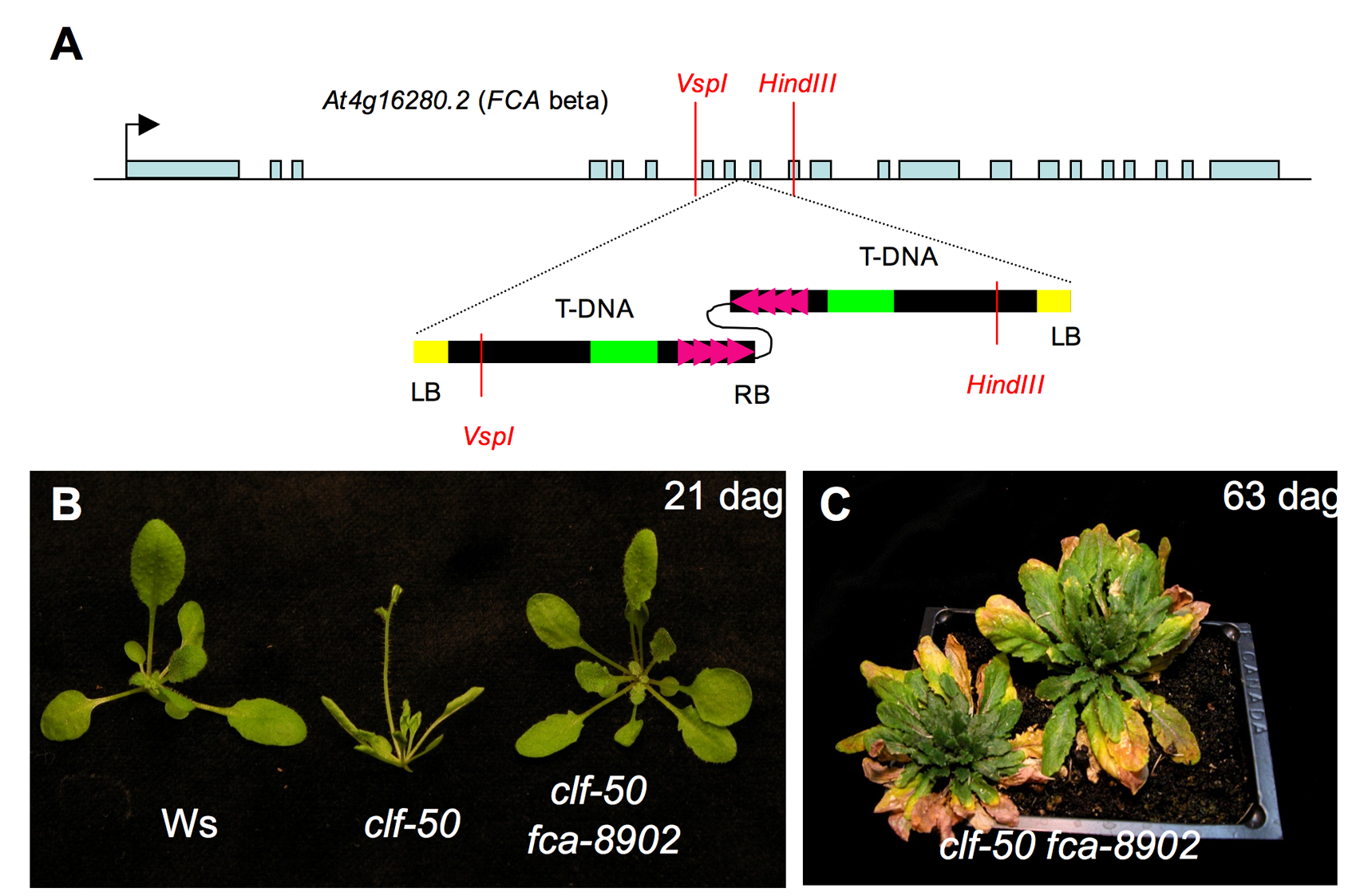

Supplement: Figure S2 — Molecular structure of fca-8902 allele. Exons are shown as light blue boxes, start of transcription indicated with an arrow. (A) fca-8902 allele. We recovered a VspI fragment and a HindIII fragment both containing T-DNA left border and plant flanking sequences. Sequence analysis of these fragments suggests a tandem insertion of at least two T DNAs in inverse orientation within the eighth intron of the FCA gene. The FCA gene produces several transcripts, the gene structure for the beta (functional) transcript is shown (B) Suppression of the early flowering and leaf curling phenotype of clf-50 by fca mutation. Long day plants 21 days after germination (dag). (C) 9 week old plants grown in long days, showing the late flowering phenotype of clf-50 fca-8902 double mutants. (TIF) [file pone.0030715.s002.tif]

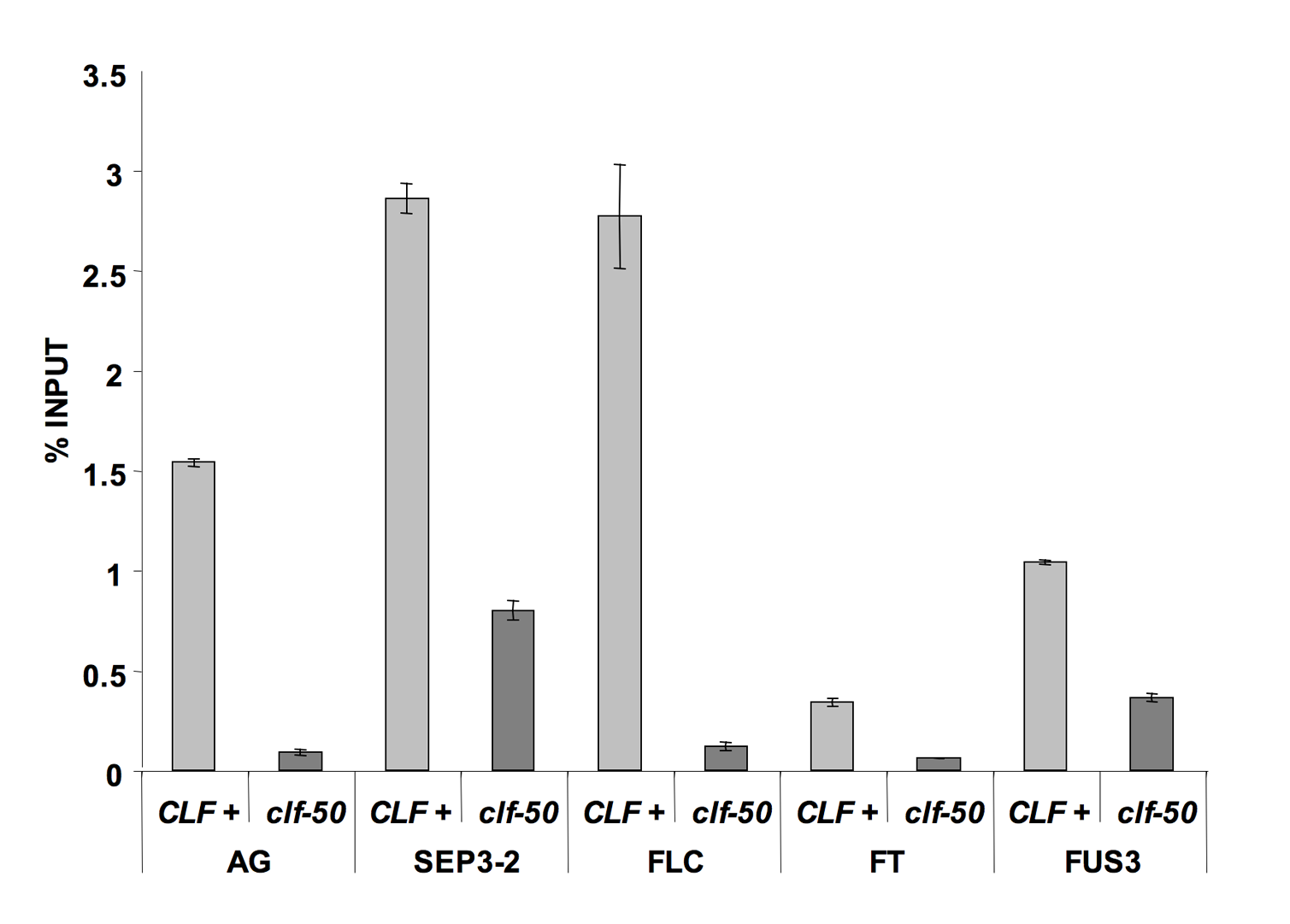

Supplement: Figure S3 — Effect of the clf mutation on histone methylation. ChIP analysis using 12 day old seedlings. Results show H3K27me3 levels at different genes, experiment was performed on independent samples from those in Figure 4. Error bars are standard error of mean of three technical replicates. (TIF) [file pone.0030715.s003.tif]
